# Cheminformatic Identification of Tyrosyl-DNA Phosphodiesterase 1 (Tdp1) Inhibitors: A Comparative Study of SMILES-Based Supervised Machine Learning Models

**DOI:** 10.3390/jpm14090981

**Published:** 2024-09-15

**Authors:** Conan Hong-Lun Lai, Alex Pak Ki Kwok, Kwong-Cheong Wong

**Affiliations:** 1 Faculty of Medicine, The Chinese University of Hong Kong, Hong Kong 999077, China; 2 Data Science and Policy Studies Programme, School of Governance and Policy Science, Faculty of Social Science, The Chinese University of Hong Kong, Hong Kong 999077, China; kwongcheongwong@cuhk.edu.hk

**Keywords:** cheminformatics, tyrosyl-DNA phosphodiesterase 1 (Tdp1), enzyme inhibition, drug identification, virtual screening, oncology, precision medicine, SMILES, machine learning

## Abstract

Background: Tyrosyl-DNA phosphodiesterase 1 (Tdp1) repairs damages in DNA induced by abortive topoisomerase 1 activity; however, maintenance of genetic integrity may sustain cellular division of neoplastic cells. It follows that Tdp1-targeting chemical inhibitors could synergize well with existing chemotherapy drugs to deny cancer growth; therefore, identification of Tdp1 inhibitors may advance precision medicine in oncology. Objective: Current computational research efforts focus primarily on molecular docking simulations, though datasets involving three-dimensional molecular structures are often hard to curate and computationally expensive to store and process. We propose the use of simplified molecular input line entry system (SMILES) chemical representations to train supervised machine learning (ML) models, aiming to predict potential Tdp1 inhibitors. Methods: An open-sourced consensus dataset containing the inhibitory activity of numerous chemicals against Tdp1 was obtained from Kaggle. Various ML algorithms were trained, ranging from simple algorithms to ensemble methods and deep neural networks. For algorithms requiring numerical data, SMILES were converted to chemical descriptors using RDKit, an open-sourced Python cheminformatics library. Results: Out of 13 optimized ML models with rigorously tuned hyperparameters, the random forest model gave the best results, yielding a receiver operating characteristics-area under curve of 0.7421, testing accuracy of 0.6815, sensitivity of 0.6444, specificity of 0.7156, precision of 0.6753, and F1 score of 0.6595. Conclusions: Ensemble methods, especially the bootstrap aggregation mechanism adopted by random forest, outperformed other ML algorithms in classifying Tdp1 inhibitors from non-inhibitors using SMILES. The discovery of Tdp1 inhibitors could unlock more treatment regimens for cancer patients, allowing for therapies tailored to the patient’s condition.

## 1. Introduction

Carcinomas, sarcomas, leukemias, lymphomas, melanomas, and other miscellaneous cancers represent a broad spectrum of malignancies that significantly impact global health. Cancers are the second leading cause of death worldwide, leading to the loss of around 4.45 million lives and 105 million disability-adjusted life years globally in 2019 alone [1]. Considerable progress has been made in oncology to combat cancers, a prominent example of which is the development and application of immunotherapies such as chimeric antigen receptor T cell (CAR-T) advanced therapy products in treating diseases, including non-Hodgkin lymphoma [2] and even some solid tumors [3]. Many promising biomarkers have also been identified for these cancer immunotherapies to target; examples include CD24 [4] and the PD-1/PD-L1 pathway [5]. Nevertheless, the diversity and complexity of cancer types pose ongoing challenges to effective treatment. The continuous emergence of new cancer variants, coupled with their heterogeneous nature, necessitates ongoing research to identify novel therapeutic strategies.

A critical area of research in this context is the identification and development of targeted therapies that can complement existing treatments and improve outcomes for cancer patients. One such promising target is tyrosyl-DNA phosphodiesterase 1 (Tdp1), an enzyme crucial for repairing DNA damage resulting from topoisomerase 1 (Top1) cleavage complexes [6,7]. Tdp1 hydrolyzes the phosphodiester bonds between DNA 3′ phosphates and Top1 tyrosine residues, thereby allowing Top1 to dissociate from its cleavage complex state and repair single-stranded breaks [8,9]. As Top1-mediated genomic lesions can disrupt DNA replication and subsequently inhibit cellular proliferation of cancer cells [10], Tdp1 inhibitors have been developed as potential cancer therapies. Tdp1 inhibitors can be used alongside Top1 inhibitors such as camptothecins and indenoisoquinoline derivatives [11,12] to prevent intentional DNA damage from being fixed by Top1 [13]. Benzylidene derivatives of usnic acid, a discovered Tdp1 inhibitor, have shown to exhibit a half-maximal inhibitory constant (IC_50_) value of 100 μM against neoplastic cells and managed to reduce tumor sizes by 62–65% [14], confirming the cancer-impeding effects of Tdp inhibitors.

Despite the promise of Tdp1 as a target for novel cancer therapies, significant research gaps persist. While ample research effort has already been dedicated to identifying Tdp1 inhibitors such as furamidine [15], CD00509 [16], and various usnic acid derivatives [15,17,18,19,20,21], they rely exclusively on physical experimentation methods of conducting biochemical assays and artificial synthesis. Though these methods have yielded valuable insights into Tdp1 inhibition, they are also incredibly resource- and manpower-demanding, rendering high-throughput screening both costly and inefficient [22]. Computational approaches offer a more viable alternative, yet their application towards the discovery of Tdp1 inhibitors has been very limited. Moshawih et al. proposed the use of three-dimensional molecular docking simulations but utilized only anthraquinone and calcone derivatives in their dataset [23], potentially restricting the scope of their findings. Similarly, Stemm’s study on designing protein-based Tdp1 inhibitors with a computation combinatorial approach [24], while innovative, faces challenges in terms of expensive synthesis and the underutilization of existing chemical compounds [25]. Bridging these gaps requires broader application of computational methods, such as high-throughput virtual screening with diverse yet accessible chemical libraries, to identify new inhibitors and advance cost-effective therapeutic strategies.

In light of these challenges, there is a growing interest in leveraging simplified molecular representations for drug discovery. The simplified molecular input line entry system (SMILES) notation, a compact and standardized representation of molecular structures using ASCII symbols, offers a less resource-intensive alternative for representing chemical structures. Literature has confirmed that SMILES representation is sufficiently reliable for quantitative structure–activity relationship (QSAR) analysis [26]. By using SMILES representations, researchers can potentially streamline the identification of Tdp1 inhibitors through machine learning (ML) models. These models, trained on chemical data represented in a SMILES format, could predict potential inhibitors with satisfactory discriminatory power, thus accelerating the drug discovery process and enhancing precision medicine approaches in oncology.

This paper aims to address the limitations of current computational methods by exploring the efficacy of SMILES-based ML models in predicting Tdp1 inhibitors. We will evaluate the use of SMILES for identifying Tdp1 inhibitors, noting that this approach represents a novel application in Tdp1 inhibitor identification. Unlike traditional biochemical methods and three-dimensional computational simulations which have been extensively explored, the use of SMILES for Tdp1 inhibitor identification has not been previously investigated. Through training a wide range of ML models, this study aims to compare and identify the most effective ML algorithms in Tdp1 inhibitor classification with SMILES. By integrating advanced ML techniques with simplified chemical representations, we hope to contribute to the advancement of targeted cancer treatments and offer new avenues for precision oncology.

## 2. Dataset and Methods

### 2.1. Data Sourcing

Isigkeit et al. curated a consensus dataset assembled from reputable databases such as ChEMBL, PubChem, and IUPHAR/BPS [27]. By using a large dataset including many general chemicals from different classes, generality could be better achieved in ML models. The original dataset indicates each chemical’s inhibition potency against drug targets by using the negative logarithm of IC_50_ values (pIC_50_), which has shown to be a better indicator of inhibition than IC_50_ values [28]. These pIC_50_ values are continuous variables, making them unsuitable for direct use in binary classification tasks. To facilitate the training of ML algorithms, pIC_50_ values greater than or equal to 6.0 [29] were assigned a new label “isActive” with a value of “1”, indicating compound activity, while values lower than 6.0 were assigned “0”, denoting inactivity. The modified dataset was obtained from Kaggle [30], and sample data from the dataset can be seen in Table 1 below.

### 2.2. Data Preprocessing

The modified dataset contains information on seven different drug targets; however, only records regarding activity towards Tdp1 are relevant to this study. Therefore, the data was filtered to include only “tdp1” under “Target”, and the “Target” column was dropped after filtering. Additionally, an unnecessary column “Unnamed: 0” containing the sequential order of records was also dropped. The filtered dataset was found to contain 1 null value under the “SMILES” column; therefore, the relevant data entry was removed.

**Figure 1:**
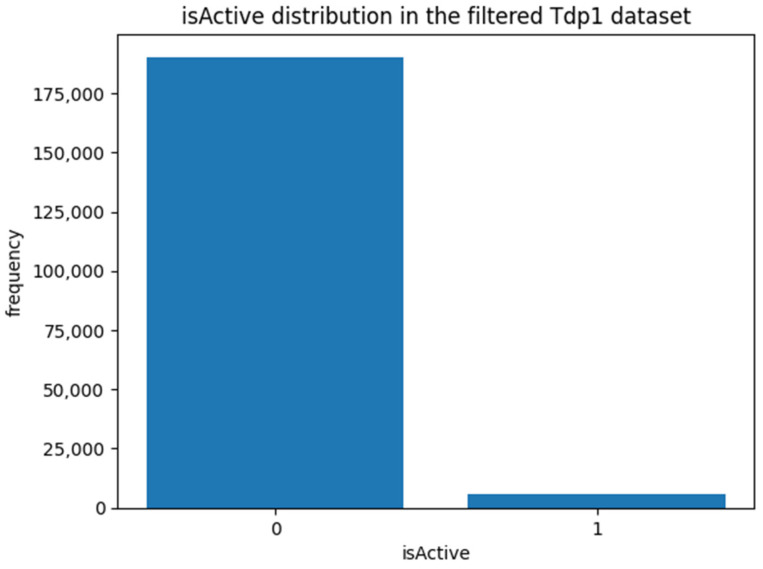
“isActive” distribution in the filtered Tdp1 dataset.

To explore whether the dataset is balanced, the number of records with “isActive” labeled 0 and 1 are computed and represented in Figure 1.

There were 190,311 records labeled 0 and 5713 records labeled 1, suggesting that the dataset is quite imbalanced. To address this imbalance, it is essential to perform downsampling of the larger class to avoid the inflated accuracy scores that may result if the ML model predictively biases towards it [31]. Randomized downsampling was carried out to ensure balanced input data to ML algorithms, where the dataset was reduced to 5713 records for both labels 0 and 1. While the viability of alternative approaches such as synthetic minority over-sampling technique (SMOTE) and cost-sensitive learning are acknowledged, randomized downsampling was preferred in this study due to its genuine and unbiased nature. For critical predictions, such as classifying potential drugs, relying on real-world data is essential. SMOTE, though effective for balancing datasets, generates synthetic examples that may fall short of capturing the true complexities of real-world data [32], and previous research has indicated that ML models trained on such synthetic data can experience performance degradation [33], a risk that is unacceptable when predicting medications with potentially life-changing consequences. Additionally, the application of cost-sensitive learning is complicated by an unclear cost structure, as the costs of type 1 (false positives) and type 2 errors (false negatives) are often comparable. While a type 1 error can lead to a waste of valuable resources for testing non-Tdp1 inhibitors with biochemical assays, a type 2 error might result in missed therapeutic opportunities, affecting patient health and treatment outcomes. This similarity in error costs reduces the practical utility of cost-sensitive methods [34]. In contrast, randomized downsampling offers a more straightforward and transparent approach, ensuring that the model’s predictions are based on authentic data and preserving the reliability and accuracy needed for high-stakes applications.

For algorithms that are unable to directly interpret text-based SMILES representation and rely on numerical input, RDKit is used for further preprocessing. RDKit is an open-source cheminformatics software available in Python capable of analyzing SMILES notation and converting it to molecular descriptors, the key features that are used to train ML algorithms [35]. The Python library “rdkit-2024.3.5” was used in this study.

A total of 208 chemical descriptors, detailed in Table S1, were returned for each SMILES string. Among these features, 12 of them contained null values as returned by RDKit, which are presented in Appendix A; therefore, they were not used when training the models. A standard scaler was also implemented to scale the descriptors [36]; then, the data were ready to be used. It is worth noting that the data were not extensively preprocessed, as the input features were provided by RDKit. It would not be ideal to modify the data excessively given that future predictions would also make use of RDKit to generate the list of input features.

### 2.3. Model Evaluation

Evaluation of the classification algorithms begin with computing the confusion matrix, which documents the way in which the classification algorithms correctly or incorrectly classifies test samples:

- True positives (TP): a true Tdp1 inhibitor correctly classified as a Tdp1 inhibitor;
- False positives (FP): a non-Tdp1 inhibitor incorrectly classified as a Tdp1 inhibitor;
- True negatives (TN): a non-Tdp1 inhibitor correctly classified as not a Tdp1 inhibitor;
- False negatives (FN): a true Tdp1 inhibitor incorrectly classified as not a Tdp1 inhibitor.

FP and FN are two erroneous metrics where the models fail to classify correctly. To better quantify the model’s classification ability, several typical metrics were used to further evaluate the models by combining the four metrics in a sensible manner. The metrics of accuracy, precision, sensitivity, specificity, F1 score, and receiver operator characteristics (ROC)-area under curve (AUC) are adopted in model evaluation [37].

Accuracy simply expresses the number of correct predictions as a percentage of the total number of predictions made. It is calculated with the following Equation (1):

(1) $$Accuracy=\frac{TP+TN}{TP+TN+FP+FN}$$

Precision refers to the percentage of predicted positive cases that were correctly classified, as shown in Equation (2):

(2) $$Precision=\frac{TP}{TP+FP}$$

Sensitivity, also known as recall or true positive rate, represents the fraction of actual positive cases that were correctly classified, as shown in Equation (3):

(3) $$Sensitivity=\frac{TP}{TP+FN}$$

Specificity, also known as true negative rate, represents the fraction of actual negative cases that were correctly classified, as shown in Equation (4):

(4) $$Specificity=\frac{TN}{TN+FP}$$

F1 score is a compact metric that is calculated by taking the harmonic mean between precision and recall. Since a trade-off often exists between precision, which represents exactness, and recall, which represents completeness, F1 score can critically assess the model’s performance in relation to these metrics, as shown in Equation (5):

(5) $$F1=\frac{2}{\frac{1}{Precision}+\frac{1}{Recall}}=\frac{2(Precision)(Recall)}{Precision+Recall}$$

ROC, as the prioritized metric in model tuning, acts as a strong indicator of the model’s ability to distinguish between the binary categories. ROC works by plotting the true positive rate against the false positive rate at different threshold settings. The ROC-AUC is often used to compare the performance of different ML models [38]:

- If ROC-AUC = 1, the model is considered perfect;
- If ROC-AUC > 0.5, the model performs better than random chance and is deemed valuable, especially when the ROC-AUC value is high;
- If ROC-AUC = 0.5, the model performs the same as random chance;
- If ROC-AUC < 0.5, the model performs worse than random chance.

The above metrics could be employed simultaneously to evaluate the predictive ability of developed ML models.

### 2.4. Machine Learning Model Building

**Figure 2:**
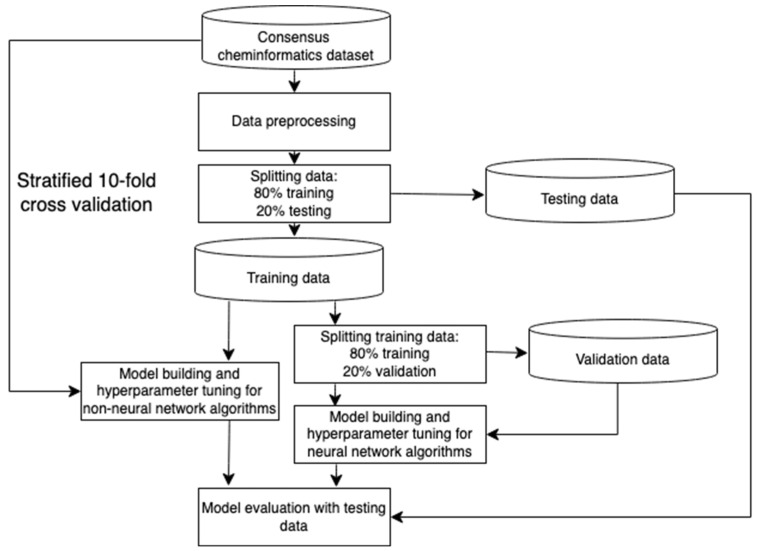
The study flow chart.

In the study flow chart shown in Figure 2, the dataset was first preprocessed and then divided, where 80% of data are used as training data and 20% are used for testing [39]. For algorithms that use numerical input data, the SMILES representation is further preprocessed with RDKit to convert to chemical descriptors. Non-neural network models were tuned using stratified 10-fold cross validation from the full dataset. For neural network models, 20% of the original training data is split off to be used as validation data [40]. Model building is promptly carried out with the use of validation data for hyperparameter tuning. After all models are built, the abovementioned evaluation metrics are then used for model evaluation.

A wide array of ML algorithms were employed in model building. Algorithms that use numerical input are the logistic regression, naïve Bayes, *k*-nearest neighbors (*k*NN), support vector machine (SVM), decision tree, random forest, gradient boosting, extreme gradient boosting (XGBoost), adaptive boosting (AdaBoost), and multilayer perceptron deep neural network (DNN). Algorithms that accept textual input are the 1-dimensional convolutional neural network (CNN), recurrent neural network (RNN), and pre-trained attention-based bidirectional encoder representations from transformers (BERTs).

As one of the simplest ML algorithms, the logistic regression algorithm performs the classification task by computing the probability *P* that the input chemical features correspond to a chemical with Tdp1-inhibitory power. By taking the inverse of the logit function to obtain the sigmoid function shown in Equation (6), a S-shaped hyperplane could be fitted across the scatter plot to compute *P* [41]:

(6) $$P=\frac{1}{1+e^{-(\beta_{0}+\sum_{i=1}^{n}x_{i}\beta_{i})}}$$

where β_0_ is the bias, and β*_i_*, *i* > 0 are the weights. They are known collectively as parameters that are “learned” by the model with training data for computing the required output. Determination of these parameters requires the use of hyperparameters specified by the ML engineer, which affects the way these parameters are learned by the algorithm. For example, the solver hyperparameter in logistic regression determines the optimization algorithm used to compute the parameters that will be used for prediction.

As derived from the Bayes theorem, the naïve Bayes algorithm computes the probability that a vector of input features *x* belongs to each of the possible output classes *y_k_* with Equation (7). In this classification task, *y*_0_ refers to non-Tdp1 inhibitor classification result, while *y*_1_ refers to Tdp1 inhibitor classification outcome.

(7) $$P(y_{k}|\vec{x})=\frac{P(\vec{x}|y_{k})P(y_{k})}{P(\vec{x})}$$

Naïve Bayes assumes that input features are independent from one another [42].

*k*NN, as the lazy learner representative, classifies test points by observing the labels of *k* number of neighbors that share the shortest distance with the point [43]. Prediction of the test point is based on the majority of neighbor labels, whether more neighbors are Tdp1 inhibitors or non-Tdp1 inhibitors. Distance is evaluated in this study using the Euclidean distance, as calculated using Equation (8):

(8) $$d(x,y)=\sqrt{\sum_{i=1}^{n}{\left(x_{i}-y_{i}\right)}^{2}}$$

*k*, the number of neighbors evaluated, represents an important hyperparameter that needs to be well selected through tuning processes. This is to avoid overfitting as a result of a low *k* value but also to avoid possible underfitting if *k* is too high.

The SVM algorithm works by attempting to fit an optimal hyperplane across the scatter plot to separate data points into different categories. The hyperplane should have the widest parallel margins, with each margin touching at least one point in each class, known as support vectors [44]. A typical hyperplane equation using the SVM algorithm is depicted in Equation (9):

(9) $$g(x)=\beta_{0}+\sum_{i=1}^{n}\vec{\beta_{i}}x_{i}$$

The decision tree algorithm attempts to split the given dataset into subsets that are dominated by a single class. The degree of impurity, or the extent to which multiple classes are mixed within the subset, is represented using entropy *S,* which is computed using Equation (10):

(10) $$S=-\sum_{i=1}^{n}p_{i}{log}_{2}p_{i}$$

where *p_i_* refers to the probability of the element belonging in the *i*^th^ subset of the parent node. The algorithm splits the dataset with attributes that cause an overall decrease in the system’s entropy. This process is then repeated with resulting child nodes until a single class dominates all child nodes, facilitating future classification efforts [45]. It is important to note that the decision tree should not be grown to maturity, as overfitting is likely to occur; therefore, a maximum depth hyperparameter should also be included before training.

As an ensemble algorithm, random forest aggregates the output of numerous base estimators by leveraging their strengths and mitigating their weaknesses. It is a robust ML algorithm characterized by its bootstrap aggregation (bagging) mechanism, which involves training numerous decision trees using bootstrap sampling of features and data entries. This approach allows each decision tree to make independent judgments based on distinct subsets of data, thereby enhancing the model’s generalization capability [46].

Boosting represents a wide variety of ML algorithms with an ensemble method, focusing on building learners to correct the mistakes of previous learners. Gradient boosting, XGBoost, and AdaBoost are popular ensemble algorithms that utilize the concept of boosting. Both Gradient Boosting and XGBoost specifically train decision trees to fit the residuals of the previous models, thereby refining predictions towards the expected output [47]. XGBoost further offers the use of incorporated regularization techniques, such as L1 (Lasso) and L2 (Ridge) regularization, which helps to prevent overfitting and improves the model’s generalization capabilities [48]. On the other hand, AdaBoost employs a rescaling technique to amplify the weights of misclassified instances. This adjustment ensures that subsequent models focus more intently on correctly classifying these higher-weighted points [49]. The predictions generated by weak learners are aggregated through a weighted voting mechanism. This weight is determined by a score assigned to each weak learner, which is computed using the log-odds function specified in Equation (11):

(11) $$score=ln(\frac{accuracy}{1-accuracy})$$

where accuracy refers to the sum of scores of correctly classified points divided by the total score of all points.

DNN is an extremely powerful class of ML algorithms as universal function approximators. The base unit of a neural network is known as the perceptron, a simple mathematical function that summates previous inputs with respect to in-built weights and biases [50]. Equation (12) shows the mathematical basis of the perceptron:

(12) $$\hat{y}=\sigma (\beta_{0}+\sum_{i=1}^{n}\beta_{i}x_{i})$$

where $\hat{y}$ represents the output of the perceptron, and σ is the activator function. Perceptrons organized in multiple dense (fully-connected) layers can be repeatedly trained through the feedforward–backpropagation mechanism to update the weights and biases connecting each perceptron [51], optimizing the model’s predictive abilities.

CNN further expands the DNN framework to include the use of convolution. Convolution involves sliding a small matrix across a given input and performing a dot product at each position to identify and highlight specific features. The convolution operation (∗) is mathematically defined in Equation (13) [52]:

(13) $$(f*g)(t)≔\int_{-\infty }^{\infty }f(\tau )g(t-\tau )d\tau$$

A 1-dimensional CNN is utilized in this study to facilitate convolution over the SMILES representation, which is simply a string of ASCII symbols.

RNNs are yet another algorithm belonging to the artificial neural network class. Unlike feedforward networks, RNNs have connections that form directed cycles, allowing them to maintain a hidden state that captures information from previous time steps [53]. RNNs are particularly suited for processing sequential data such as text; thus, it is appropriate to investigate its ability to interpret SMILES. Since traditional RNNs may suffer from the vanishing gradient problem, which hampers their ability to learn from long-range dependencies, the gated recurrent unit (GRU) architecture is adopted, as this problem is addressed by its gating mechanism [54]. While the long short-term memory (LSTM) architecture is a viable alternative, GRU is chosen due to its simplicity and need for less hyperparameter tuning while maintaining comparable computational efficacy and performance.

Transformers, the most recent addition to the artificial neural network family, remain the most crucial advancement in ML. Introduced by Vaswani et al. in 2017, transformers leverage a self-attention mechanism to capture context dependencies among tokens. This significantly improves contemporary computational abilities in interpreting text-based unstructured data, especially since homophones can be distinguished and interpreted with contextual understanding. The attention mechanism can be mathematically understood using Equation (14) [55]:

(14) $$Attention(Q,K,V)=softmax(\frac{QK^{T}}{\sqrt{d_{k}}})V$$

where *Q*, *K*, *V* refer to the query, key, and value matrices, respectively, *T* refers to the length of the input sequence, and *d_k_* refers to the dimension of the particular key–query space. BERT employs the transformer neural architecture and further boasts bidirectionality: the ability to examine both left-to-right and right-to-left contextual dependencies [56]. Capability for bidirectional attention analysis renders BERT a suitable transformer model for interpreting chemical formulas that possess holistic structure–function relations, justifying its use in this study.

It is important to note that these ML algorithms often rely on numerous assumptions, such as data linearity, homogeneity, as well as the independent and identical distribution of variables [57]. However, previous reviews have shown that the ML algorithms mentioned above have shown potential in drug discovery [58,59], justifying the choice of algorithms used here. Non-neural network models were built using the scikit-learn library “sklearn-1.3.2”, except for the XGBoost model, which was built with “xgboost-2.1.1”. Neural network models were built using the tensorflow library “tensorflow-2.17.0” in Python. The BERT model was built using the PyTorch “torch-2.3.1 + cu121” and transformers “transformers-4.42.4” libraries.

**Figure 3:**
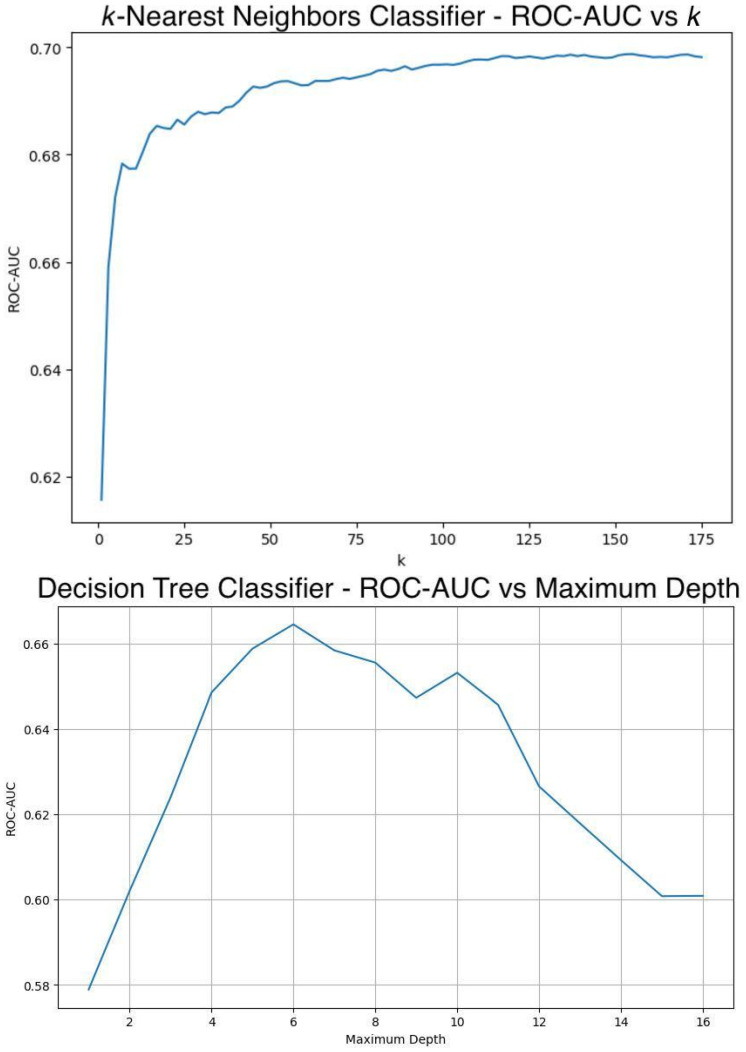
Tuning respective hyperparameters for *k*-nearest neighbors (*k*NN) and decision tree classifiers by exhaustion.

**Figure 4:**
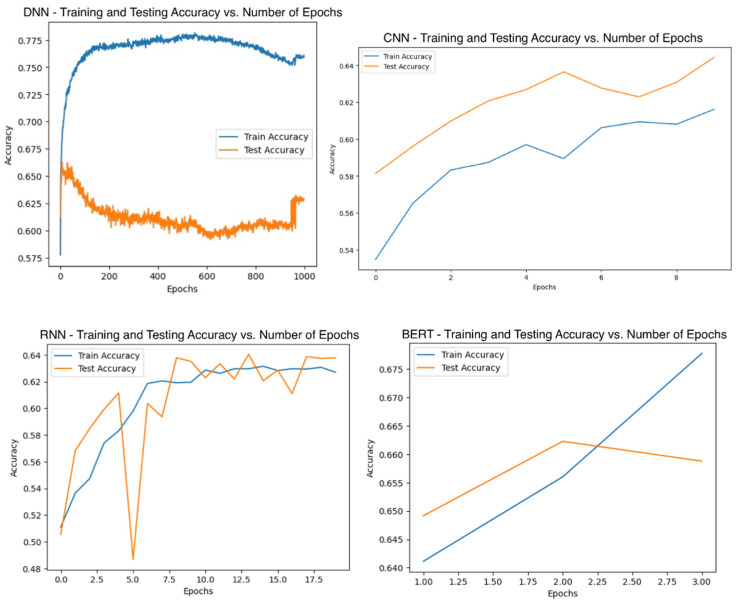
Finding the optimal number of epochs for neural network models by comparing training and testing accuracy to avoid underfitting or overfitting.

ROC-AUC is the key metric in optimizing ML models because it effectively demonstrates the model’s capacity to differentiate between Tdp1 inhibitors and non-inhibitors [38]. Therefore, hyperparameter values or combinations that gave the highest ROC-AUC score upon tuning were selected. Hyperparameters were rigorously tuned with strict validation steps: for all non-neural network algorithms, stratified 10-fold cross validation with the full set of data was used to ensure that hyperparameters chosen are not affected by any implicit biases inflicted by the random train-test splitting of data [60]. An exhaustive method was adopted to tune ML algorithms with only one hyperparameter, such as *k*NN and decision tree. Hyperparameter values yielding models with the highest ROC-AUC were adopted, as seen in Figure 3. In algorithms where more than one hyperparameter needs to be tuned, such as SVM and AdaBoost, Bayesian optimization was used alongside stratified 10-fold cross validation to search for the best combination to be used. Bayesian optimization guides the search towards promising regions as informed by previous search results, acting as an efficient optimizer for expensive-to-evaluate models [61]. The library “bayesian-optimization-1.5.1”, along with the supplementary package “colorama-0.4.6” for coloured text printing, were used in this study. For all neural network algorithms, including BERT, a separate set of validation data was used for model tuning. To determine the neural architecture, a neural architecture search (NAS) [62] was first carried out to get a general idea of the approximate best architecture, and then manual tuning was carried out to optimize the architecture, as detailed in Appendix B. The learning rate was tuned by adopting the “reduce learning rate on plateau” mechanism: once the ROC-AUC has stopped improving, the learning rate is reduced [63]. The optimal number of epochs was tuned by an exhaustive search by continuously running the feedforward–backpropagation steps, evaluating the ROC-AUC after each epoch, and saving only the best model with the highest ROC-AUC. Graphs of training and validation accuracy against number of epochs were also drawn in the end to ensure the selected number of epochs did not create an overfitted model [64], as shown in Figure 4. Other hyperparameters, such as filter size and kernel size, were tuned using grid search. Table S2 details the hyperparameter search space, while Table 2 shows all hyperparameters used after the tuning process.

## 3. Results

Table 3 below displays the evaluation metrics obtained from the optimized ML models, correct to four significant figures.

**Figure 5:**
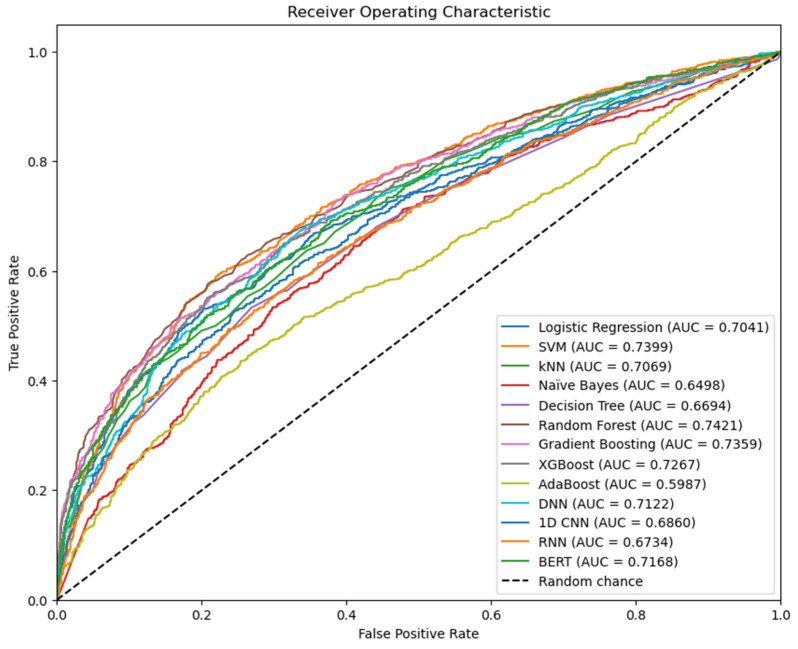
Combined ROC curves for all optimized ML algorithms.

ROC-AUC is the prioritized metric in this study due to its ability to indicate the model’s ability to gauge differences between Tdp1 inhibitors and non-inhibitors. Figure 5 combines all ROC curves together into one graph for comparison.

The random forest model is undoubtedly the best model developed by this study, as it achieved the highest accuracy (0.6815), sensitivity (0.6444), F1 score (0.6595), and ROC-AUC (0.7421) out of all models. Naïve Bayes had the best specificity (0.9195), while XGBoost achieved the highest precision (0.7776). Most ML models developed in this study demonstrate a satisfactory ability in classifying Tdp1 inhibitors from non-inhibitors as all ROC-AUC values exceed 0.5, indicating that their classifying ability performs better than random chance.

Random forest is a powerful ML algorithm owing to its bagging mechanism: it trains a large number of decision trees, each with stochastically selected features and data entries through bootstrap sampling. As a result, all decision trees can make independent judgements based on their unique features and datasets, enabling a strong generalization ability [65]. The superior performance of the random forest model in this study, having an ROC-AUC of 0.7421, suggests that its ensemble method by bagging is particularly effective in interpreting complex molecular features and relating them to Tdp1 inhibition. Since random forest is only one of the possible bagging methods, other bagging algorithms involving a different base estimator could be further explored for their potential in drug discovery.

On the other hand, some ML models face difficulty in the classification task. It is interesting to note that the worst model across most metrics was AdaBoost, with an exceptionally low ROC-AUC (0.5987). It may be possible that the combined voting classifier poses a source of error for this model. Naïve Bayes has a particularly low sensitivity (0.2038) and F1 score (0.3156), which may be due to its assumption of independence between input features. As chemical features are likely to be correlated in the same chemical, the failure of this “naive” algorithm to recognize the inapplicability of its assumption is bound to impact its identification ability.

## 4. Discussion

### 4.1. Practical Implications

The classification model developed in this study serves as a powerful tool for drug discovery. Prediction of potential Tdp1 inhibitors enables researchers to focus their efforts on the most promising compounds, streamlining the drug development process [66]. This targeted approach can significantly accelerate the identification of effective inhibitors, potentially leading to new therapeutic options for cancer patients.

Additionally, our model could contribute to knowledge generation through reverse engineering. If a compound is identified as having a significant effect on Tdp1 that was previously undocumented, this finding could spur further research to elucidate its mechanism of action [67]. Such insights are valuable for understanding how different compounds interact with biological targets, which can inform future drug design and discovery efforts.

Moreover, a significant benefit of utilizing ML models for Tdp1 inhibitor identification lies in its computational methodology, which obviates the need for physical chemical experimentation at the initial stage. Virtual screening provides a rapid, sustainable, and resource-efficient method of discovering potential Tdp1 inhibitors, as compared to the extensive use of biochemical assays [68]. Though the development of ML models requires ample time and computational power, subsequent predictions could be achieved in a matter of milliseconds upon input of SMILES chemical representations, all without employing further chemical resources. When screening of chemical compounds is required in high volumes for drug discovery, the use of virtual screening with developed ML models is greatly advantageous over the conventional experimental methods of high-throughput screening.

This study’s methodology leveraged a large, general dataset of chemical compounds, which offers several advantages. The use of a broad dataset ensures the generality and applicability of our model across a wide range of chemicals, enhancing the robustness of the predictions [69]. This approach allows for the evaluation of diverse chemical structures, which is essential in identifying novel inhibitors that may not have been previously considered.

One of the significant strengths of this study’s approach is the use of accessible input data—SMILES representation—instead of more complex and computationally intensive molecular structures. SMILES provides a convenient and efficient means of representing molecular structures [70], which simplifies the data preparation process and reduces computational overhead. This accessibility makes the method readily applicable in various settings, including those with limited computational resources. ML models developed in this study allow for the rapid and scalable screening of diverse chemical compounds, facilitating the discovery of personalized therapeutic options tailored to individual patients’ molecular profiles. The ability to predict and prioritize compounds that specifically interact with Tdp1 opens new avenues for developing targeted therapies [71], which are essential for personalizing treatment regimens based on the unique genetic and biochemical characteristics of each patient. Thus, this study’s method supports the advancement of precision medicine by enabling the identification of drugs that can be customized to effectively address individual patients’ needs.

Importantly, while our cheminformatic model offers a rapid and preliminary means of identifying potential inhibitors, it is crucial to integrate these predictions with established methods to ensure safety and efficacy. This study’s approach is designed to complement traditional techniques such as molecular docking, assay tests, and clinical trials. These proven methods provide validation and confirmation of the predictions made by our model, ensuring that the identified compounds are not only promising but also safe for further development [72,73].

### 4.2. Limitations and Future Work

There are several methods available for hyperparameter tuning, each with its own advantages and limitations. In addition to Bayesian optimization, two other prevalent techniques are grid search and randomized search. In this study, the Bayesian optimization algorithm was utilized due to its efficiency in navigating the hyperparameter space, as well as its capability to balance exploration and exploitation through a probabilistic model. This approach is particularly advantageous when dealing with complex, high-dimensional parameter spaces, as it systematically identifies promising hyperparameter configurations while minimizing computational costs [74]. In contrast, grid search involves an exhaustive evaluation of predefined hyperparameter combinations, which can be computationally prohibitive with large search spaces. On the other hand, randomized search samples hyperparameter configurations randomly, which is much more computationally efficient but does not ensure full coverage of the search space [75]. Bayesian optimization thus serves as a middle ground, combining the systematic approach of grid search with the efficiency of randomized search. It effectively navigates the hyperparameter space by prioritizing the exploration of promising regions while avoiding the exhaustive computational demands of grid search, thereby optimizing performance with fewer iterations. While there is no perfect method in hyperparameter optimization, this study adopts a balanced approach with Bayesian optimization which strikes a compromise between result optimization and computational burden. Further research efforts could investigate the use of different tuning methods to find more optimized hyperparameter combinations.

Additionally, while our study demonstrated the efficacy of supervised ML algorithms for discriminating Tdp1 inhibitors from non-inhibitors, the individual contributions of each specific component to the ML models were not thoroughly considered. To address this gap, future work could incorporate ablation studies to systematically assess the impact of different features and model elements on predictive performance. Such analyses should focus on assessing the impact of specific components of the ML models, such as model architectures, hyperparameter settings, and training strategies [76]. By evaluating the significance of each component, future research can improve the robustness and interpretability of the ML models, leading to more effective identification of potential inhibitors.

In the context of interpreting unstructured medical data using ML, particularly with natural language processing (NLP) techniques, effective tokenization strategies are crucial [77]. For genomic data, there is a well-established consensus to use the *k*-mers technique, where *k* is a multiple of three, aligning with the codon structure of genetic sequences that are read in triplets of bases [78]. However, for SMILES representations of chemical compounds, an optimal tokenization approach has yet to be universally agreed upon. Currently, the conventional method involves character-level tokenization, as employed in this study. Alternative methods include the *k*-mers technique, atom-level tokenization, and SMILES substring encoding, where tokenization is based on functional groups. The latter, being the most recent and potentially superior approach, represents a promising advancement in the field [79]. Future research on the use of SMILES for drug discovery with combined NLP–ML approaches could employ the use of functional group encoding to better represent how groups of atoms work together meaningfully to shape the molecule’s physicochemical profile, implicating how it interacts with different drug targets.

In addition, if the purpose of developing ML models is for analytical purposes such as knowledge discovery, the use of regression models can be considered. By predicting the pIC_50_ each chemical exhibits on a drug target, it could assist reverse-engineering efforts to pinpoint the mechanism by which inhibitors interact with drug targets, facilitating more nuanced insights. Other unsupervised learning methods, such as principal component analysis, could provide further insight into the most important physicochemical features which affect its interaction with drug targets [80]. On the other hand, for identifying plausible inhibitors, a classification model remains a practical choice. This method is not only adequate for determining potential inhibitors but also tends to yield results that are more straightforward to interpret.

Future work should focus on leveraging the ML models developed in this study to identify potential Tdp1 inhibitors. These models, having demonstrated promise in predicting inhibitor candidates, can be further utilized to generate a comprehensive list of plausible inhibitors. Subsequent experimental validation will be crucial to confirm the efficacy of these candidates. Physical experimental techniques, such as enzyme assays and structural binding studies [81], should be employed to verify that the inhibitors identified through ML models indeed exhibit Tdp1 inhibitory activity. This integration of computational predictions with experimental verification will not only enhance the reliability of the identified inhibitors, but also contribute to the broader goal of developing effective and targeted cancer therapies [82].

Furthermore, the effectiveness of the random forest algorithm in identifying Tdp1 inhibitors from SMILES data suggests promising avenues for future research. To enhance the predictive performance and broaden the applicability of these models, future work could involve investigating alternative base estimators within the bagging framework. While decision trees used by random forests have proven effective, exploring other base estimators—such as SVM, *k*NN, perceptrons, or a mix of several [83]—could offer additional insights and improve the model’s capability to interpret and analyze SMILES representations. By systematically evaluating and comparing these alternative base estimators, researchers can optimize the bagging approach and potentially uncover novel patterns and relationships in chemical data. This exploration may lead to more robust and accurate identification of Tdp1 inhibitors and further advance the application of computational methods in drug discovery.

Future research could also focus on enhancing the interpretability of the ML models developed in this study, thereby making the classification process more transparent and comprehensible for human analysts. Given that certain chemical features may be more informative than others in predicting Tdp1-inhibiting abilities, incorporating advanced feature importance analysis methods is crucial. Techniques such as principal component analysis (PCA) and t-distributed stochastic neighbor embedding (t-SNE) could be employed to elucidate the relative significance of different features [84]. Furthermore, the integration of Shapley additive explanation (SHAP) values could provide additional insights into feature contributions by offering a unified measure of feature importance that accounts for interactions between features [85]. Future studies could improve model interpretability by leveraging these approaches, enhancing the practical applicability and explainability of ML classifications in predicting Tdp1 inhibitors.

Additionally, in the age of large language models (LLMs), the use of LLMs could also be exploited in cheminformatic data mining. Studies have shown that LLMs such as generative pre-trained transformers and Large Language Model Meta AI (LLaMA) are capable of interpreting SMILES notions and predicting drug–drug interactions [86]. Although applications of LLM are beyond the scope of this study, alternative methods of interpreting SMILES notation could be considered for future research.

## 5. Conclusions

This study demonstrates that the integration of cheminformatics with ML can play a significant role in identifying potential Tdp1 inhibitors from non-inhibitors with SMILES representation. By using a large dataset with a wide range of chemical classes offering generalizability, the random forest model developed here shows promising results in predicting chemical compounds with Tdp1 inhibiting power. With an ROC-AUC of 0.7421, testing accuracy of 0.6815, sensitivity of 0.6444, specificity of 0.7156, precision of 0.6753, and F1 score of 0.6595, the random forest model shows satisfying predictive ability in classifying Tdp1 inhibitors with its ensemble bagging algorithm. Models developed by this study could serve as an initial filter to discern chemicals with Tdp1-inhibiting abilities, giving room for further specific tests such as molecular docking and clinical trials to take place. This allows for the safe, efficient identification of Tdp1 inhibitors, paving the way for personalized oncology treatments in the future by suppressing cancer growth.

## Figures and Tables

**Table 1:** Sample data from the dataset.

Unnamed: 0	SMILES	Target	isActive
493,977	O=C1C(Cc2ccc3[nH]ccc3c2)=NC(=S)N1c1ccccc1	tdp1	0
493,978	O=C(Nc1cc(C(F)(F)F)cc2c1SSSSS2)C(F)(F)F	tdp1	0
493,979	CCOC(=O)c1[nH]nc2c1C(=O)c1ncccc1C2=O	tdp1	1
493,980	O=C1NC(=O)C(=Cc2ccc3[nH]ccc3c2)S1	tdp1	0
493,981	O=C1NC(=S)C(=Cc2ccc3[nH]ccc3c2)S1	tdp1	0

**Table 2:** Hyperparameters adopted for ML after tuning.

Model	Hyperparameter (s)
Logistic regression	solver: limited-memory Broyden–Fletcher–Goldfarb–Shanno algorithm (LBFGS)
Naïve Bayes	Gaussian naïve Bayes
*k*NN	*k* (number of nearest neighbors): 155
SVM	kernel: radial basis function (RBF) C (penalty parameter): 10^0.0762^ gamma: 10^−2.0529^
Decision tree	maximum depth: 6
Random forest	number of estimators: 415 maximum features: 3 maximum depth: 50
Gradient boosting	number of estimators: 341 maximum depth: 7 learning rate: 0.01
XGBoost	objective: binary: logitraw number of estimators: 293 maximum depth: 10 learning rate: 0.01
AdaBoost	estimator: voting classifier, combining: logistic regression with LBFGS solver; Gaussian naïve Bayes, and; decision tree with maximum depth 1. number of estimators: 487 learning rate: 0.1989
DNN	neural architecture (Figure S1): Input: 198; Dense: 10 [activator = Rectified Linear Unit (ReLU)]; Dense: 4 [activator = ReLU]; Dense: 1 [activator = sigmoid]. optimiser: root mean square propagation (RMSprop) loss function: binary cross entropy epochs: 13
CNN (1D)	tokenization: character level neural architecture (Figure S2): Embedding [output: 128]; Convolutional 1D [filter: 64, kernel size: 5, activator: ReLU, L2 regularization: 0.001]; Global maximum pooling 1D; Dropout [dropout rate: 0.3]; Dense: 10 [activator: ReLU, L2 regularization: 0.001]; Dropout [dropout rate: 0.3]; Dense: 1 [activator: sigmoid]. optimizer: adaptive moment estimation (Adam) loss function: binary cross entropy batch size: 32 epochs: 10
RNN (GRU)	tokenization: character level neural architecture (Figure S3): Embedding [output: 128]; Bidirectional gated recurrent unit (GRU): 128; Dropout [dropout rate: 0.5]; Bidirectional GRU: 128; Dropout [dropout rate: 0.5]; Dense: 64 [activator: ReLU]; Dropout [dropout rate: 0.5]; Dense: 1 [activator: sigmoid]. optimizer: RMSprop (learning rate: 2e^−4^) loss function: binary cross entropy batch size: 32 epochs: 18
BERT	pre-trained BERT tokenizer and model: bert-base-uncased neural architecture of bert-base-uncased (Figure S4) optimizer: AdamW (learning rate = 2e^−5^) epochs: 3

**Table 3:** Evaluating the performance of optimized ML models.

Model	Accuracy	Sensitivity	Specificity	Precision	F1 Score	ROC-AUC
Logistic regression	0.6562	0.6051	0.7030	0.6516	0.6275	0.7041
Naïve Bayes	0.5770	0.2038	0.9195	0.6991	0.3156	0.6498
*k*NN	0.6457	0.5155	0.7651	0.6682	0.5820	0.7069
SVM	0.6763	0.6298	0.7198	0.6732	0.6503	0.7399
Decision tree	0.6282	0.5631	0.6879	0.6235	0.5917	0.6694
Random forest	0.6815	0.6444	0.7156	0.6753	0.6595	0.7421
Gradient boosting	0.6697	0.6335	0.7030	0.6619	0.6474	0.7359
XGBoost	0.6514	0.3803	0.9002	0.7776	0.5107	0.7267
AdaBoost	0.5836	0.5229	0.6393	0.5709	0.5458	0.5987
DNN	0.6649	0.6060	0.7190	0.6643	0.6338	0.7122
CNN (1D)	0.6426	0.4717	0.7995	0.6934	0.5581	0.6860
RNN (GRU)	0.6295	0.5046	0.7441	0.6441	0.5659	0.6734
BERT	0.6557	0.5320	0.7693	0.6791	0.5966	0.7168

## Data Availability

The dataset used to support the findings of this study was derived from Prototype Chemgenomics Dataset available at Kaggle at https://www.kaggle.com/datasets/williamtbarker/prototype-chemgenomics-dataset (accessed on 5 June 2024).

## Supplementary Materials

The following supporting information can be downloaded at: https://www.mdpi.com/article/10.3390/jpm14090981/s1, Figure S1: Deep neural network (DNN) neural architecture; Figure S2: Convolutional neural network (CNN) neural architecture; Figure S3: Recurrent neural network (RNN) neural architecture; Figure S4: Bidirectional encoder representations from transformer (BERT) neural architecture; Table S1: List of chemical descriptors and fragments used in RDKit. Table S2: Hyperparameter search space. Supplementary code: Computer code in Python used to train the machine learning (ML) models.

## Appendix A

**Table A1:** Chemical descriptors returned by RDKit containing null values.

RDKit Variable Name	Descriptor Full Name	Description
MaxPartialCharge	Maximum partial charge	Maximum partial charge found on any atom in the molecule.
MinPartialCharge	Minimum partial charge	Minimum partial charge found on any atom in the molecule.
MaxAbsPartialCharge	Maximum absolute partial charge	Maximum absolute value of partial charge on any atom.
MinAbsPartialCharge	Minimum absolute partial charge	Minimum absolute value of partial charge on any atom.
BCUT2D_MWHI	BCUT2D maximum weight by atom	Maximum value of atomic weights in the molecule according to burden-centered descriptor (BCUT2D) analysis.
BCUT2D_MWLOW	BCUT2D minimum weight by atom	Minimum value of atomic weights in the molecule according to BCUT2D analysis.
BCUT2D_CHGHI	BCUT2D maximum charge	Maximum charge value associated with the atoms in the molecule according to BCUT2D analysis.
BCUT2D_CHGLO	BCUT2D minimum charge	Minimum charge value associated with the atoms in the molecule according to BCUT2D analysis.
BCUT2D_LOGPHI	BCUT2D maximum logP	Maximum value of the logarithm of the partition coefficient (logP)Maximum charge value associated with the atoms in the molecule according to BCUT2D analysis, reflecting hydrophobicity
BCUT2D_LOGPLOW	BCUT2D minimum logP	Minimum value of logP according to BCUT2D analysis, indicating the lowest hydrophobicity.
BCUT2D_MRHI	BCUT2D maximum refractivity	Maximum refractivity value among the atoms in the molecule according to BCUT2D analysis.
BCUT2D_MRLOW	BCUT2D minimum refractivity	Minimum refractivity value in the molecule according to BCUT2D analysis.

## Appendix B

**Table A2:** List of tested deep neural network (DNN) architectures with best number of epochs and corresponding ROC-AUC scores.

Trial Number	Tested Architecture	Optimal Number of Epochs	ROC-AUC Score
1	198 (input) × 4 × 4 × 1 (output)	2	0.6961
2	198 (input) × 4 × 2 × 1 (output)	2	0.6961
3	198 (input) × 8 × 1 × 1 (output)	22	0.7071
4	198 (input) × 10 × 1 × 1 (output)	10	0.7001
5	198 (input) × 10 × 2 × 1 (output)	13	0.7010
6	198 (input) × 8 × 2 × 1 (output)	33	0.7088
7	198 (input) × 10 × 4 × 1 (output)	13	0.7122
8	198 (input) × 10 × 5 × 1 (output)	7	0.7088
9	198 (input) × 10 × 3 × 1 (output)	19	0.7058
