# Supplementary material for: Cheminformatic Identification of Tyrosyl-DNA Phosphodiesterase 1 (Tdp1) Inhibitors: A Comparative Study of SMILES-Based Supervised Machine Learning Models
Source: J Pers Med. 2024 Sep 15;14(9):981. doi: 10.3390/jpm14090981 (PMC11433629; doi:10.3390/jpm14090981)
Supplement: Supplementary file 1 [file jpm-14-00981-s001.zip › Table S1.pdf]

Table S1. List of chemical descriptors and fragments used in RDKit.

S/N	RDKit Variable Name	Descriptor Full Name	Description
1	MaxEStateIndex	Maximum electrotopological state index	Highest value of electrotopological state indices among all atoms in the molecule.
2	MinEStateIndex	Minimum electrotopological state index	Lowest value of electrotopological state indices among all atoms in the molecule.
3	MaxAbsEStateIndex	Maximum absolute electrotopological state index	Maximum absolute value of electrotopological state indices across all atoms.
4	MinAbsEStateIndex	Minimum absolute electrotopological state index	Minimum absolute value of electrotopological state indices across all atoms.
5	qed	Quantitative estimate of drug-likeness	Estimates how "drug-like" a molecule is based on properties such as lipophilicity and molecular weight.
6	MolWt	Molecular weight	Total weight of all atoms in a molecule, calculated as the sum of the atomic weights of all constituent atoms.
7	HeavyAtomMolWt	Heavy atom molecular weight	Molecular weight of a molecule calculated excluding hydrogen atoms, reflecting weight from heavier elements.
8	ExactMolWt	Exact molecular weight	Precise weight of a molecule based on the exact isotopic masses of its constituent atoms.
9	NumValenceElectrons	Number of valence electrons	Total number of valence electrons in a molecule available for bonding.
10	NumRadicalElectron	Number of radical electrons	Number of unpaired electrons or radicals in the molecule, indicating its radical character.
11	MaxPartialCharge	Maximum partial charge	Maximum partial charge found on any atom in the molecule.
12	MinPartialCharge	Minimum partial charge	Minimum partial charge on any atom in the molecule.

13	MaxAbsPartialCharge	Maximum absolute partial charge	Maximum absolute value of partial charge on any atom.
14	MinAbsPartialCharge	Minimum absolute partial charge	Minimum absolute value of partial charge on any atom.
15	FpDensityMorgan1	Fingerprint density Morgan 1	Density measure of the Morgan fingerprint with radius 1, indicating the distribution of substructures.
16	FpDensityMorgan2	Fingerprint density Morgan 2	Density measure of the Morgan fingerprint with radius 2, reflecting the distribution of larger substructures.
17	FpDensityMorgan3	Fingerprint density Morgan 3	Density measure of the Morgan fingerprint with radius 3, showing the distribution of even larger substructures.
18	BCUT2D_MWHI	BCUT2D maximum weight by atom	Maximum value of atomic weights in the molecule according to burden-centered descriptor (BCUT2D) analysis.
19	BCUT2D_MWLOW	BCUT2D minimum weight by atom	Minimum value of atomic weights in the molecule according to BCUT2D analysis.
20	BCUT2D_CHGHI	BCUT2D maximum charge	Maximum charge value associated with the atoms in the molecule according to BCUT2D analysis.
21	BCUT2D_CHGLO	BCUT2D minimum charge	Minimum charge value observed in the atoms of the molecule according to BCUT2D analysis.
22	BCUT2D_LOGPHI	BCUT2D maximum logP	Maximum value of the logarithm of the partition coefficient (logP) according to BCUT2D analysis, reflecting hydrophobicity.
23	BCUT2D_LOGPLOW	BCUT2D minimum logP	Minimum value of logP according to BCUT2D analysis, indicating the lowest hydrophobicity.
24	BCUT2D_MRHI	BCUT2D maximum refractivity	Maximum refractivity value among the atoms in the molecule according to BCUT2D analysis.
25	BCUT2D_MRLOW	BCUT2D minimum refractivity	Minimum refractivity value in the molecule according to BCUT2D analysis.

26	BalabanJ	Balaban's J index	A topological index quantifying molecular complexity based on atom connectivity.
27	BertzCT	Bertz's complexity index	Measures molecular complexity based on unique atom types and connectivity.
28	Chi0	Chi 0 index	A topological descriptor related to the number of vertices (atoms) in a molecule.
29	Chi0n	Chi 0 index (normal)	A normalized version of the Chi 0 Index, providing a standardized measure of molecular structure.
30	Chi0v	Chi 0 index (vertex)	Variation of the Chi 0 Index that considers vertex contributions.
31	Chi1	Chi 1 index	A topological descriptor related to the number of paths of length 1 between pairs of vertices.
32	Chi1n	Chi 1 index (normal)	A normalized version of the Chi 1 Index, providing a standardized measure of molecular connectivity.
33	Chi1v	Chi 1 index (vertex)	Variation of the Chi 1 Index that includes vertex contributions.
34	Chi2n	Chi 2 index (normal)	A normalized version of the Chi 2 Index, related to paths of length 2 between vertices.
35	Chi2v	Chi 2 index (vertex)	Variation of the Chi 2 Index that considers vertex contributions.
36	Chi3n	Chi 3 index (normal)	A normalized version of the Chi 3 Index, related to paths of length 3 between vertices.
37	Chi3v	Chi 3 index (vertex)	Variation of the Chi 3 Index that includes vertex contributions.
38	Chi4n	Chi 4 index (normal)	A normalized version of the Chi 4 Index, related to paths of length 4 between vertices.
39	Chi4v	Chi 4 index (vertex)	Variation of the Chi 4 Index that considers vertex contributions.

40	HallKierAlpha	Hall-Kier Alpha index	A topological index measuring molecular complexity based on ring size and connectivity.
41	Ipc	Intrinsic path count	A measure of the number of independent paths in the molecular graph, reflecting connectivity complexity.
42	Kappa1	Kappa 1 index	A topological descriptor that reflects the connectivity of atoms in a molecule, specifically focusing on the Kappa 1 value.
43	Kappa2	Kappa 2 index	A topological descriptor similar to Kappa 1 but with different weightings, indicating molecular connectivity.
44	Kappa3	Kappa 3 index	A topological descriptor that provides further detail on the connectivity of atoms, focusing on the Kappa 3 value.
45	LabuteASA	Labute's atomic surface area	Surface area accessible to a solvent, which can be important for understanding molecular interactions.
46	PEOE_VSA1	PEOE VSA 1	Van der Waals surface area (VSA) descriptors in the partial equalization of orbital electronegativity (PEOE) series, reflecting specific types of surface areas.
47	PEOE_VSA10	PEOE VSA 10	
48	PEOE_VSA11	PEOE VSA 11	
49	PEOE_VSA12	PEOE VSA 12	
50	PEOE_VSA13	PEOE VSA 13	
51	PEOE_VSA14	PEOE VSA 14	
52	PEOE_VSA2	PEOE VSA 2	
53	PEOE_VSA3	PEOE VSA 3	
54	PEOE_VSA4	PEOE VSA 4	
55	PEOE_VSA5	PEOE VSA 5	

56	PEOE_VSA6	PEOE VSA 6	
57	PEOE_VSA7	PEOE VSA 7	
58	PEOE_VSA8	PEOE VSA 8	
59	PEOE_VSA9	PEOE VSA 9	
60	SMR_VSA1	SMR VSA 1	VSA descriptors derived using the sum of mean refractivity (SMR) framework, also measuring different aspects of surface areas.
61	SMR_VSA10	SMR VSA 10	
62	SMR_VSA2	SMR VSA 2	
63	SMR_VSA3	SMR VSA 3	
64	SMR_VSA4	SMR VSA 4	
65	SMR_VSA5	SMR VSA 5	
66	SMR_VSA6	SMR VSA 6	
67	SMR_VSA7	SMR VSA 7	
68	SMR_VSA8	SMR VSA 8	
69	SMR_VSA9	SMR VSA 9	
70	SlogP_VSA1	SlogP VSA 1	VSA descriptors adhering to the logarithm of partition coefficient (SlogP) framework, reflecting specific surface areas related to SlogP values.
71	SlogP_VSA10	SlogP VSA 10	
72	SlogP_VSA11	SlogP VSA 11	
73	SlogP_VSA12	SlogP VSA 12	
74	SlogP_VSA2	SlogP VSA 2	

75	SlogP_VSA3	SlogP VSA 3	
76	SlogP_VSA4	SlogP VSA 4	
77	SlogP_VSA5	SlogP VSA 5	
78	SlogP_VSA6	SlogP VSA 6	
79	SlogP_VSA7	SlogP VSA 7	
80	SlogP_VSA8	SlogP VSA 8	
81	SlogP_VSA9	SlogP VSA 9	
82	TPSA	Topological polar surface area	Total polar surface area of a molecule, relevant for understanding its interactions with solvents.
83	EState_VSA1	Electrotopological state VSA 1	VSA descriptors based on electrotopological states, reflecting particular surface area measurements.
84	EState_VSA10	Electrotopological state VSA 10	
85	EState_VSA11	Electrotopological state VSA 11	
86	EState_VSA2	Electrotopological state VSA 2	
87	EState_VSA3	Electrotopological state VSA 3	
88	EState_VSA4	Electrotopological state VSA 4	
89	EState_VSA5	Electrotopological state VSA 5	
90	EState_VSA6	Electrotopological state VSA 6	
91	EState_VSA7	Electrotopological state VSA 7	

92	EState_VSA8	Electrotopological state VSA 8	
93	EState_VSA9	Electrotopological state VSA 9	
94	VSA_EState1	VSA electrotopological state 1	Electrotopological state descriptors based on VSA, showing specific electrotopological state values.
95	VSA_EState10	VSA electrotopological state 10	
96	VSA_EState2	VSA electrotopological state 2	
97	VSA_EState3	VSA electrotopological state 3	
98	VSA_EState4	VSA electrotopological state 4	
99	VSA_EState5	VSA electrotopological state 5	
100	VSA_EState6	VSA electrotopological state 6	
101	VSA_EState7	VSA electrotopological state 7	
102	VSA_EState8	VSA electrotopological state 8	
103	VSA_EState9	VSA electrotopological state 9	
104	FractionCSP3	Fraction of sp^3 hybridized carbons	Proportion of carbon atoms in a molecule that are sp^3 hybridized, indicating the level of saturation.
105	HeavyAtomCount	Heavy atom count	Number of non-hydrogen atoms in a molecule, reflecting its overall size and complexity.
106	NHOHCount	Number of amine (NH) or hydroxyl (OH) Groups	The count of amine or hydroxyl groups in the molecule, relevant for understanding its functional groups.

107	NOCount	Number of nitrogen (N) or oxygen (O) atoms	The count of nitrogens or hydrogens in the molecule, relevant for understanding its functional groups.
108	NumAlphaticCarbocycles	Number of aliphatic carbocycles	Count of aliphatic carbocyclic rings in the molecule, indicating cyclic structures with single bonds.
109	NumAliphaticHeterocycles	Number of aliphatic heterocycles	Number of aliphatic heterocyclic rings, showing the presence of rings with heteroatoms and single bonds.
110	NumAliphaticRings	Number of aliphatic rings	Total count of aliphatic rings, reflecting the cyclic nature and saturation of the molecule.
111	NumAromaticCarbocycles	Number of aromatic carbocycles	Number of aromatic carbocyclic rings, indicating the presence of stable, conjugated rings.
112	NumAromaticHeterocycles	Number of aromatic heterocycles	Count of aromatic heterocyclic rings, showing the presence of conjugated rings with heteroatoms.
113	NumAromaticRings	Number of aromatic rings	Total number of aromatic rings, indicating the degree of aromaticity in the molecule.
114	NumHAcceptors	Number of hydrogen bond acceptors	Count of atoms that can accept hydrogen bonds, such as oxygen or nitrogen atoms.
115	NumHDonors	Number of hydrogen bond donors	Number of atoms capable of donating hydrogen bonds, typically including hydroxyl or amino groups.
116	NumHeteroatoms	Number of heteroatoms	Count of atoms in the molecule that are not carbon, usually including nitrogen, oxygen, sulfur, etc.
117	NumRotatableBonds	Number of rotatable bonds	Number of bonds in the molecule that can rotate freely, influencing flexibility and conformation.
118	NumSaturatedCarbocycles	Number of saturated carbocycles	Count of saturated carbocyclic rings, indicating cyclic structures with single bonds only.
119	NumSaturatedHeterocycles	Number of saturated heterocycles	Number of saturated heterocyclic rings, reflecting the presence of rings with single bonds and heteroatoms.

120	NumSaturatedRings	Number of saturated rings	Total count of saturated rings in the molecule, showing cyclic structures without double bonds.
121	RingCount	Ring count	Total number of rings in the molecule, reflecting its cyclic structure complexity.
122	MolLogP	Molecular logP	Logarithm of the partition coefficient (logP), indicating the hydrophobicity of the molecule.
123	MolMR	Molecular refractivity	Overall refractivity of the molecule, related to its polarizability.
124	fr_Al_COO	Number of aliphatic carboxylic acid	Count of aliphatic carboxyl groups (COO) in the molecule.
125	fr_Al_OH	Number of aliphatic hydroxyl groups	Count of aliphatic hydroxyl groups (OH) in the molecule.
126	fr_Al_OH_noTert	Number of aliphatic hydroxyl groups excluding tertiary alcohols	Count of aliphatic hydroxyl groups (OH), excluding tertiary alcohols, in the molecule.
127	fr_ArN	Number of nitrogen functional groups attached to aromatics	Count of nitrogen functional groups such as amines, amides, nitriles, etc., attached to aromatic rings
128	fr_Ar_COO	Number of aromatic carboxylic acid	Count of aromatic carboxylic acid groups (COO) in the molecule.
129	fr_Ar_N	Number of aromatic nitrogens	Count of nitrogens (N) in an aromatic ring.
130	fr_Al_NH	Number of aromatic amines	Count of amine groups (NH) in an aromatic ring.
131	fr_Al_OH	Number of aromatic hydroxyl groups	Count of hydroxyl groups (OH) in an aromatic ring.
132	fr_COO	Number of carbon-attached carboxyl groups	Count of carboxyl groups (COO) attached to carbons.
133	fr_COO2	Number of carboxylic acids	Count of carboxylic acid groups (COOH) in the molecule.

134	fr_C_O	Number of carbonyl oxygens	Count of carbonyl oxygen groups (C=O) in the molecule.
135	fr_C_O_noCOO	Number of carbonyl oxygens except carboxylic acids	Count of carbonyl oxygen groups (C=O), excluding carboxylic acid groups (COO), in the molecule.
136	fr_C_S	Number of thiocarbonyl groups	Count of thiocarbonyl groups (C=S) in the molecule.
137	fr_HOCCN	Number of C(OH)CCN groups	Count of C(OH)CCN groups, especially in the form C(OH)CCN-C(tertiary alkyl) or C(OH)CCN(cyclic structure).
138	fr_Imine	Number of imine groups	Count of imine groups (C=S) in the molecule.
139	fr_NH0	Number of tertiary amine groups	Count of tertiary amine groups (R_3N) in the molecule.
140	fr_NH1	Number of secondary amine groups	Count of secondary amine groups (R_2NH) in the molecule.
141	fr_NH2	Number of primary amine groups	Count of primary amine groups (RNH_2) in the molecule.
142	fr_N_O	Number of hydroxylamines	Count of hydroxylamines (NH_2OH) in the molecule.
143	fr_Ndealkylation1	Number of XCCNR groups	Count of XCNNR groups which are typically involved in N-dealkylation reactions.
144	fr_Ndealkylation2	Number of tert-alicyclic amine groups	Count of tert-alicyclic amine groups, without including heteroatoms or quinine-like bridged nitrogen structures.
145	fr_Nhpyrrole	Number of H-pyrrole nitrogens	Count of H-pyrrole nitrogens, which are nitrogen atoms within a pyrrole ring that have a hydrogen atom attached to them.
146	fr_SH	Number of thiol groups	Count of thiol groups (SH) in the molecule.
147	fr_aldehyde	Number of aldehydes	Count of aldehyde groups (R_2CH=O) in the molecule.
148	fr_alkyl_carbamate	Number of alkyl carbamate groups	Count of carbamate groups ($R_2NC(O)OR$) attached to alkyl groups, subject to

			hydrolysis.
149	fr_alkyl_halide	Number of alkyl halides	Count of halides attached to alkyl groups in the molecule.
150	fr_allylic_oxid	Number of allylic oxidation sites	Count of allylic oxidation sites, excluding steroid dienone, in the molecule.
151	fr_amide	Number of amide groups	Count of amide groups ($RC(=O)-NR_2$) in the molecule.
152	fr_amidine	Number of amidine groups	Count of amidine groups ($RC(NR)NR_2$) in the molecule.
153	fr_aniline	Number of anilines	Count of anilines ($C_6H_5NH_2$) in the molecule.
154	fr_aryl_methyl	Number of aryl methyl sites	Count of aryl methyl sites available for hydroxylation.
155	fr_azide	Number of azide groups	Count of azide groups (RN_3) in the molecule.
156	fr_azo	Number of azo groups	Count of azo groups (R-N=N-R) in the molecule.
157	fr_barbitur	Number of barbiturate groups	Count of barbiturate groups in the molecule.
158	fr_benzene	Number of benzene rings	Count of benzene rings in the molecule.
159	fr_benzodiazepine	Number of benzodiazepine groups	Count of benzodiazepine groups ($C_9H_8N_2$), with no additional fused rings, in the molecule.
160	fr_bicyclic	Number of bicyclic rings	Count of fused or bridged bicyclic rings in the molecule.
161	fr_diazo	Number of diazo groups	Count of diazo groups ($R_2C=N=N$) in the molecule.
162	fr_dihydropyridine	Number of dihydropyridines	Count of dihydropyridines ($CH_2(HC=CH)_2NH$) in the molecule.
163	fr_epoxide	Number of epoxide rings	Count of epoxide rings (cyclic R_2C-R_2C-O-) in the molecule.
164	fr_ester	Number of ester groups	Count of ester groups (RCOOR) in the molecule.

165	fr_ether	Number of ether oxygens	Count of oxygens in ether groups (R-O-R), including the phenoxy group (C_6H_5OR)
166	fr_furan	Number of furan rings	Count of furan rings in the molecule.
167	fr_guanido	Number of guanidine groups	Count of guanidine groups in the molecule.
168	fr_halogen	Number of halogens	Count of halogens such as fluorine, chlorine, bromine, etc. in the molecule.
169	fr_hdrzine	Number of hydrazine groups	Count of hydrazine groups (R_2N-NR_2) in the molecule.
170	fr_hdrzone	Number of hydrazone groups	Count of hydrazone groups (R_2C=N-NH_2) in the molecule.
171	fr_imidazole	Number of imidazole rings	Count of imidazole rings in the molecule.
172	fr_imide	Number of imide groups	Count of imide groups in the molecule.
173	fr_isocyan	Number of isocyanate groups	Count of isocyanate groups (RN=C=O) in the molecule.
174	fr_isothiocyan	Number of isothiocyanate groups	Count of isothiocyanate groups (RN=C=S) in the molecule.
175	fr_ketone	Number of ketones	Count of ketone groups (RC(=O)R) in the molecule.
176	fr_ketone_Topliss	Number of ketones, excluding diaryl, ab,-unsat, dienones, heteroatom on Calpha	Count of ketone groups (RC(=O)R), excluding diaryl, α,β-unsaturated acids, dienones, or heteroatoms on C_α.
177	fr_lactam	Number of beta-lactam rings	Count of beta-lactam rings in the molecule.
178	fr_lactone	Number of cyclic esters (lactones)	Count of cyclic esters, such as lactones, in the molecule
179	fr_methoxy	Number of methoxy groups	Count of methoxy groups ($ROCH_3$) in the molecule.
180	fr_morpholine	Number of morpholine rings	Count of morpholine rings in the molecule.

181	fr_nitrile	Number of nitrile groups	Count of nitrile groups (RC≡N) in the molecule.
182	fr_nitro	Number of nitro groups	Count of nitro groups (RNO_2) in the molecule.
183	fr_nitro_arom	Number of nitro benzene rings substituents	Count of nitrobenzene ($C_6H_5NO_2$) in the molecule.
184	fr_nitro_arom_nonortho	Number of non–ortho benzene ring substituents	Count of non-ortho benzene ring substituents in the molecule.
185	fr_nitroso	Number of nitroso groups	Count of nitroso groups (RN=O) in the molecule, except NO_2.
186	fr_oxazole	Number of oxazole rings	Count of oxazole rings in the molecule.
187	fr_oxime	Number of oxime groups	Count of oxime groups (R_2C=NOH) in the molecule.
188	fr_para_hydroxylation	Number of para-hydroxylation sites	Count of total para-hydroxylation sites in the molecule.
189	fr_phenol	Number of phenol groups	Count of phenol groups in the molecule.
190	fr_phenol_noOrthoHBond	Number of phenol groups, excluding ortho intramolecular H bond substituents	Count of phenol groups, excluding ortho intramolecular hydrogen bond substituents.
191	fr_phos_acid	Number of phosphoric acid groups	Count of phosphoric acid groups in the molecule.
192	fr_phos_ester	Number of phosphoric ester groups	Count of phosphoric ester groups in the molecule.
193	fr_piperdine	Number of piperdine rings	Count of piperdine rings in the molecule.
194	fr_piperzine	Number of piperzine rings	Count of piperzine rings in the molecule.
195	fr_priamide	Number of primary amide groups	Count of primary amide groups ($RC(=O)NH_2$) in the molecule.

196	fr_prisulfonamd	Number of primary sulfonamides	Count of primary sulfonamide groups (R-SO_2NR_2) in the molecule.
197	fr_pyridine	Number of pyridine rings	Count of pyridine rings (C_5H_5N) in the molecule.
198	fr_quatN	Number of quaternary nitrogens	Count of quaternary nitrogens (NR_4^+) in the molecule.
199	fr_sulfide	Number of thioether groups	Count of thioether groups (RSR) in the molecule.
200	fr_sulfonamd	Number of sulfonamide groups	Count of sulfonamide groups in the molecule.
201	fr_sulfone	Number of sulfone groups	Count of sulfone groups ($R\text{-}S(=O)_2R$) in the molecule.
202	fr_term_acetylene	Number of terminal acetylene groups	Count of terminal acetylene groups (RC≡CH) in the molecule.
203	fr_tetrazole	Number of tetrazole rings	Count of tetrazole rings in the molecule.
204	fr_thiazole	Number of thiazole rings	Count of thiazole rings in the molecule.
205	fr_thiocyan	Number of thiocyanate groups	Count of thiocyanate groups (RSC≡N) in the molecule.
206	fr_thiophene	Number of thiophene rings	Count of thiophene rings in the molecule.
207	fr_unbrch_alkane	Number of unbranched alkanes of at least 4 members	Count of unbranched alkanes with at least 4 or more members, excluding halogenated alkanes.
208	fr_urea	Number of urea groups	Count of urea groups in the molecule.
