# Supplementary material for: Cheminformatic Identification of Tyrosyl-DNA Phosphodiesterase 1 (Tdp1) Inhibitors: A Comparative Study of SMILES-Based Supervised Machine Learning Models
Source: J Pers Med. 2024 Sep 15;14(9):981. doi: 10.3390/jpm14090981 (PMC11433629; doi:10.3390/jpm14090981)
Supplement: Supplementary file 1 [file jpm-14-00981-s001.zip › Table S2.pdf]

Table S2. Hyperparameter search space.

Model	Hyperparameter(s) searched
Logistic regression	solvers:<br>• Newton's method with conjugate gradient descent (Newton-CG);<br>• Newton's method with Cholesky factorization (Newton-Cholesky);<br>• Limited-memory Broyden-Fletcher-Goldfarb-Shanno algorithm (LBFGS)<br>• Library for large linear classification (LibLinear)<br>• Stochastic average gradient (SAG), and;<br>• Stochastic average gradient accelerated (SAGA).
Naïve Bayes	Gaussian naïve Bayes only: Multinomial and Bernoulli naïve Bayes could not be used as some input features are binary and some are continuous.
*k*NN	*k* (number of nearest neighbors): Odd positive integers starting from 1, stop when ROC-AUC does not increase after 10 consecutive increments of *k*.
SVM	kernels:<br>• Linear;<br>• Sigmoid, and;<br>• Radial basis function (RBF).<br>C (penalty parameter), applicable to sigmoid and RBF kernels only: from 10^{-3} to 10^{3}.<br>gamma, applicable to sigmoid and RBF kernels only: from 10^{-3} to 10^{3}.
Decision tree	maximum depth: Positive integers starting from 1, stop when ROC-AUC does not increase after 10 consecutive increments of maximum depth.
Random forest	number of estimators: integers from 100 to 500.<br>maximum features: integers from 1 to 10.<br>maximum depth: integers from 1 to 50.
Gradient boosting	number of estimators: integers from 100 to 500.<br>maximum depth: integers from 1 to 10<br>learning rate: floating point numbers from 0.01 to 0.9.
XGBoost	objectives:<br>• Binary:logistic;<br>• Binary:hinge, and;<br>• Binary:logitraw.<br>number of estimators: integers from 100 to 500.<br>maximum depth: integers from 1 to 10.<br>learning rate: floating point numbers from 0.01 to 0.9.
AdaBoost	estimators:<br>• Logistic regression with LBFGS solver;<br>• Gaussian naïve Bayes;<br>• Decision tree with maximum depth 1, and;<br>• A voting classifier combining all three.<br>number of estimators: integers from 50 to 500.

	learning rate: floating point numbers from 0.01 to 0.9.
DNN	neural architecture:<br>● Number of layers: 1, 2, 3, 4, 5.<br>● Number of neurons per layer: 1, 2, 4, 8, 16, 32, 64.<br>optimizers:<br>● Stochastic gradient descent (SGD);<br>● Root mean square propagation (RMSprop);<br>● Adaptive gradient algorithm (AdaGrad), and;<br>● Adaptive moment estimation (Adam).<br>epochs: positive integers from 1 to 1000.
CNN (1D)	filter: 64, 128.<br>kernel size: 3, 5.<br>dropout rates = 0.3, 0.5.<br>L2 regularization: 0.001, 0.00001.<br>batch size: 32, 64.<br>epochs: positive integers from 1 to 10.
RNN (GRU)	initial learning rate: 0.001, tuned using the “reduce learning rate on plateau” function,<br>minimum learning rate: 0.0001.<br>epochs: positive integers from 1 to 20.
