# Supplementary figures and images for: Cheminformatic Identification of Tyrosyl-DNA Phosphodiesterase 1 (Tdp1) Inhibitors: A Comparative Study of SMILES-Based Supervised Machine Learning Models
Source: J Pers Med. 2024 Sep 15;14(9):981. doi: 10.3390/jpm14090981 (PMC11433629; doi:10.3390/jpm14090981)

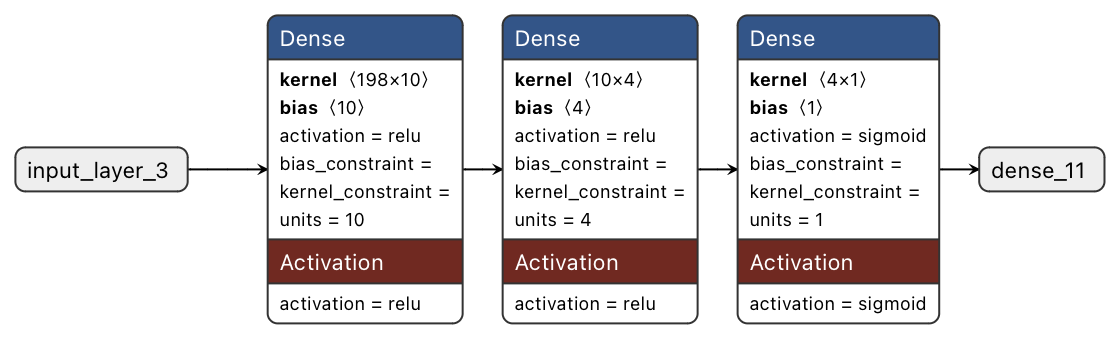

Supplement: Supplementary file 1 [file jpm-14-00981-s001.zip › Figure S1.png]

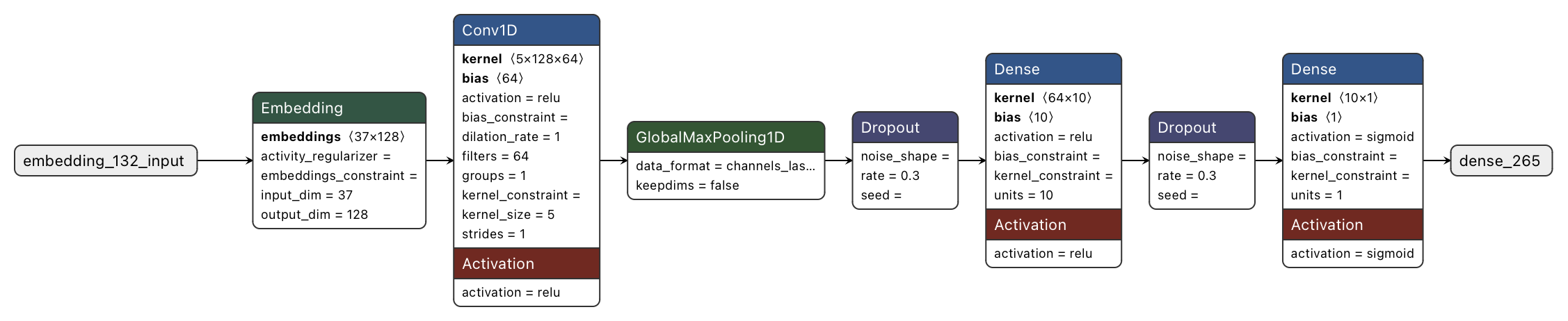

Supplement: Supplementary file 1 [file jpm-14-00981-s001.zip › Figure S2.png]

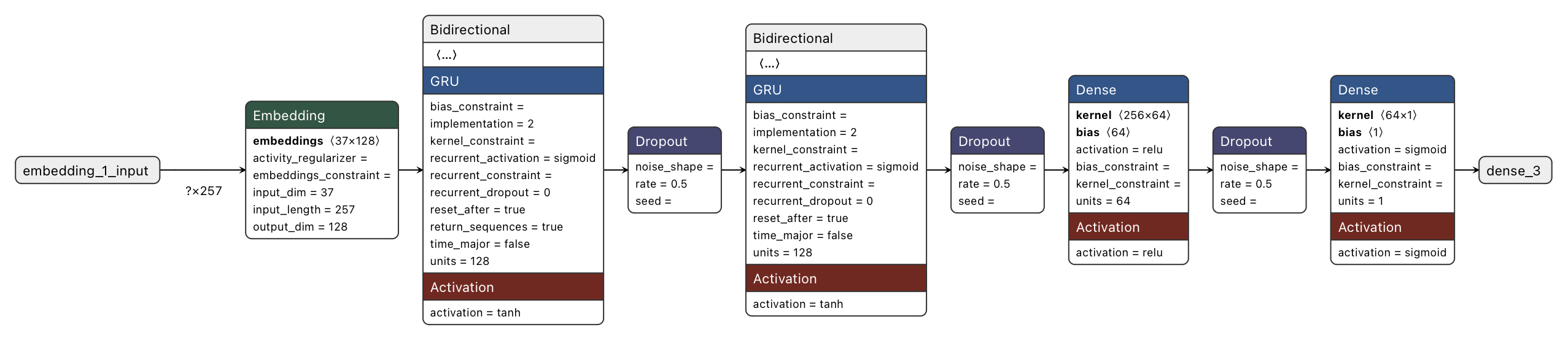

Supplement: Supplementary file 1 [file jpm-14-00981-s001.zip › Figure S3.png]
